# Supplementary material for: The Astragalus Membranaceus Herb Attenuates Leukemia by Inhibiting the FLI1 Oncogene and Enhancing Anti-Tumor Immunity
Source: Int J Mol Sci. 2024 Dec 14;25(24):13426. doi: 10.3390/ijms252413426 (PMC11676164; doi:10.3390/ijms252413426)
Supplement: Supplementary file 1 [file ijms-25-13426-s001.zip › ijms-3338275-supplementary.pdf]

Supplemental Material

1. Supplemental Tables

Table S1 RT-q-PCR gene primers sequence.

Gene	Forward	Reverse
GAPDH	GGAGCGAGATCCCTCCAAAAT	GGCTGTTGTCATACTTCTCATGG
FLI1	CCAACGAGAGGAGAGTCATCG	CCAACGAGAGGAGAGTCATCG
GATA1	TTGTCAGTAAACGGGCAGGTA	CTTGCGGTTTCGAGTCTGAAT
IFN-γ	TCGGTAACTGACTTGAATGTCCA	TCGCTTCCCTGTTTTAGCTGC
TDO2	TCCTCAGGCTATCACTACCTGC	ATCTTCGGTATCCAGTGTCGG
IDO1	GCCAGCTTCGAGAAAGAGTTG	ATCCCAGAACTAGACGTGCAA
HDC	ATGCACGCCTACTACCCAG	CAGTCCATGACGTTCATCTCC
TNFa	CCTCTCTCTAATCAGCCCTCTG	GAGGACCTGGGAGTAGATGAG
TNFAIP3	TCCTCAGGCTTTGTATTTGAGC	TGTGTATCGGTGCATGGTTTTA
IL1B	ATGATGGCTTATTACAGTGGCAA	GTCGGAGATTCGTAGCTGGA
IL1A	TGGTAGTAGCAACCAACGGGA	ACTTTGATTGAGGGCGTCATTC
IL6	ACTCACCTCTTCAGAACGAATTG CC	ATCTTTGGAAGGTTCAGGTTG
CXCR2	CCTGTCTTACTTTTCCGAAGGAC	TTGCTGTATTGTTGCCCATGT

Table S2 87 compounds in AM from TCMSP

Mol ID	Molecule Name	Mol ID	Molecule Name
MOL000114	vanillic acid	MOL000389	Ferulic acid
MOL000131	EIC	MOL000390	daidzein
MOL001955	Heriguard	MOL000391	Ononin
MOL000251	Rhamnocitrin	MOL000393	Soyasaponin I
MOL000295	alexandrin	MOL000394	choline
MOL000356	lupeol	MOL000395	GGB
MOL000372	3-Hydroxy-2-picoline	MOL000396	(+)-Syringaresinol
MOL000373	(2S)-4-methoxy-7-methyl-2-[1-methyl-1-[(2S,3R,4S,5S,6R)-3,4,5-trihydroxy-6-methylol-tetrahydropyran-2-yl]oxy-ethyl]-2,3-dihydrofuro[3,2-g]chromen-5-one	MOL000033	(3S,8S,9S,10R,13R,14S,17R)-10,13-dimethyl-17-[(2R,5S)-5-propan-2-yloctan-2-yl]-2,3,4,7,8,9,11,12,14,15,16,17-dodecahydro-1H-cyclopenta[a]phenanthren-3-ol
MOL000375	5′_hydroxyiso_muronulatol_2′_5′_di_O_glucoside_qt	MOL000438	(3R)-3-(2-Hydroxy-3,4-dimethoxyphenyl)chroman-7-ol
MOL000376	7,2'-dihydroxy-3',4'-dimethoxyisoflavone-7-O-β-D-glucoside	MOL000380	(6aR,11aR)-9,10-Dimethoxy-6a,11a-dihydro-6H-benzofurano[3,2-c]chromen-3-ol
MOL000377	7-hydroxy-3-(2-hydroxy-3,4-dimethoxy-phenyl)chromone	MOL000436	(Z)-1-(2,4-dihydroxyphenyl)-3-(4-hydroxyphenyl)prop-2-en-1-one
MOL000381	13_hydroxy_9_11_octadecadienoic acid	MOL000400	Flavaxin
MOL000382	Arabinose	MOL000401	astragalosideI
MOL000383	D_Galacturonic acid_ homopolymer	MOL000402	astragalosideI_qt

MOL000386	Fucopyranose, L-	MOL000397	cis-p-Coumarate
MOL000388	gamma-aminobutyric acid	MOL000069	palmitic acid
MOL000384	DL_Glucuronic acid	MOL005928	isoferulic acid
MOL000387	Bifendate	MOL000399	Docosanoate
MOL000392	Formononetin	MOL000371	3,9-di-O-methylnissolin
MOL000398	Isoflavanone	MOL000211	Betulinic acid（Mairin）
MOL000417	Calycosin	MOL000239	Jaranol
MOL000422	Kaempferol	MOL000433	FA
MOL000403	astragalosideⅡ	MOL000423	rhamnocitrin-3-O-glucoside
MOL000404	astragalosideⅡ_qt	MOL000424	RAM
MOL000405	astragalosideⅢ	MOL000425	asernestioside A
MOL000406	astragalosideⅢ_qt	MOL000426	asernestioside A_qt
MOL000409	astragalosideⅣ	MOL000427	asernestioside B
MOL000410	astragalosideⅣ_qt	MOL000428	asernestioside B_qt
MOL000411	Astraisoflavanin	MOL000429	Crystal VI
MOL000418	3'_Hydroxy_4'_methoxyisoflavone_7_O_beta_D_glucoside	MOL000296	Hederagenin
MOL000378	7-O-methylisomucronulatol	MOL000379	9,10-Dimethoxypterocarpan-3-O-β-D-glucoside
MOL000440	isomucronulatol-7,2'-di-O-glucosiole_qt	MOL000374	5'-Hydroxyiso-muronulatol-2',5'-di-O-glucoside
MOL000439	Isomucronulatol-7,2'-di-O-glucosiole	MOL000442	1,7-Dihydroxy-3,9-dimethoxypterocarpene
MOL000413	astrachrysoside A	MOL000430	Betaine
MOL000415	rutin	MOL000431	coumarin
MOL000416	Lariciresinol	MOL000432	linolenic acid
MOL000412	Mucronulatol	MOL000434	acetylastragaloside I
MOL000414	Caffeate	MOL000435	acetylastragaloside I_qt
MOL000420	XLS	MOL000437	Hirsutrin
MOL000421	Nicotinic acid	MOL000441	LUPENONE
MOL000407	AstragalosideⅣ	MOL000061	Prolinum
MOL000408	AstragalosideⅣ_qt	MOL000054	L-Arginin
MOL000419	astrasieversianin XV	MOL000354	Isorhamnetin
MOL000098	Quercetin		

Table S3 The chemical components in Ai identified by UPLC-MS/MS

Number	Compounds name	Number	Compounds name
1	6,10-bis(acetyloxy)-2-ethenyl-4,10a-dihydroxy-2,4b,8,8-tetramethyl-7-oxo-1,2,3,4,4a,4b,7,8,8a,9,10,10a-dodecahydrophenanthren-1-yl acetate	91	3,8,9,10-tetrahydroxy-2-(hydroxymethyl)-6-oxo-1H,2H,3H,4H,4aH,6H,10bH-benzo[c]chromen-4-yl 4-hydroxy-3,5-dimethoxybenzoate
2	1,7-dihydroxy-4-methoxy-1-(2-oxopropyl)-1,2-dihydrophenanthren-2-one	92	6-hydroxy-1-(hydroxymethyl)-6-methyl-1H,2H,6H,7H,8H,9H,10H,11H-phenanthro[1,2-b]furan-10,11-dione
3	4a-hydroxy-6a-methyl-2-oxo-1a,2,4a,4b,5,6,6a,7,8,9,9a,9b,10,11-tetradecahydrocyclopenta[7,8]phenanthro[1,10a-b]oxiren-7-yl acetate	93	1-{3a,7-dihydroxy-9a,11a-dimethyl-1H,2H,3H,3aH,3bH,4H,6H,7H,8H,9H,9aH,9bH,10H,11H,11aH-

			cyclopenta[a]phenanthren-1-yl}-2-methoxyethan-1-one
4	6-(9-hydroxy-2a,5a,8,8-tetramethyltetradecahydro-1H,12H-cyclopenta[a]cyclopropa[e]phenanthren-3-yl)-2-methylhept-2-enal	94	8-hydroxy-1-(hydroxymethyl)-7-isopropyl-1,4a-dimethyl-3,4,4a,9,10,10a-hexahydrophenanthren-2(1H)-one
5	7-hydroxy-6-(hydroxymethyl)-1-methyl-3bH,6H,7H,8H,9H,9bH,10H,11H-phenanthro[1,2-b]furan-10,11-dione	95	7-ethenyl-5a-hydroxy-7,9b-dimethyl-1H,3H,3bH,4H,5H,5aH,6H,7H,8H,9H,9aH,9bH,10H,11H-phenanthro[1,2-c]furan-1-one
6	6,10-bis(acetyloxy)-2-ethenyl-4,10a-dihydroxy-2,4b,8,8-tetramethyl-7-oxo-1,2,3,4,4a,4b,7,8,8a,9,10,10a-dodecahydrophenanthren-1-yl acetate	96	7,11-dihydroxy-4-(hydroxymethyl)-3,9,11b-trimethyl-1H,2H,6H,11bH-phenanthro[3,2-b]furan-2,6-dione
7	1,5-dihydroxy-1,9a,11a-trimethyl-1H,2H,3H,3aH,3bH,4H,5H,7H,9aH,9bH,10H,11H,11aH-cyclopenta[a]phenanthren-7-one	97	3b,4-dihydroxy-1,6,6,9a-tetramethyl-hexadecahydrophenanthro[1,2-b]furan-2,11-dione
8	1-hydroxy-1,9a,11a-trimethyl-1H,2H,3H,3aH,3bH,7H,8H,9H,9aH,9bH,10H,11H,11aH-cyclopenta[a]phenanthren-7-one	98	4a,6a-dimethyl-3,4,4a,6,6a,8,9,9a,9b,10-decahydro-2H-dispiro[indeno[5,4-f]chromene-7,4'-[1,3]dioxolane-5',4''-[1,3]dioxolane]-2,5(4bH)-dione
9	1,3,4-trimethoxy-2,7-dihydrophenanthrene-2,7-dione	99	(3R,4S,5S,17R,20R)-3,17,20-Trihydroxy-4-Methylpregn-8-En-7-One
10	8-(hydroxymethyl)-9a,11a-dimethyl-hexadecahydro-1H-cyclopenta[a]phenanthrene-1,7-diol	100	(5aS,9S,9aR,9bS)-9-hydroxy-5a,9-dimethyl-4,5,9a,9b-tetrahydro-3aH-benzo[g][1]benzofuran-2,3,6-trione
11	6,6-dimethyl-10,11-dioxo-1H,2H,6H,7H,8H,9H,10H,11H-phenanthro[1,2-b]furan-1-carboxylic acid	101	7-hydroxy-6-(hydroxymethyl)-1-methyl-3bH,6H,7H,8H,9H,9bH,10H,11H-phenanthro[1,2-b]furan-10,11-dione
12	1-ethenyl-5a,7-dihydroxy-9a,11a-dimethyl-hexadecahydro-1H-cyclopenta[a]phenanthren-5-one	102	1-ethynyl-1-hydroxy-11a-methyl-hexadecahydro-1H-cyclopenta[a]phenanthren-7-one
13	4-(8,11-dihydroxy-4a,4b,7,7,10a-pentamethyl-2-methylene-1,2,3,4,4a,4b,5,6,6a,7,8,9,10,10a,10b,11-hexadecahydrochrysen-1-yl)-3-methylpentanoic acid	103	5a-hydroxy-9a,11a-dimethyl-1-(6-methyl-5-methylideneheptan-2-yl)-7-oxo-hexadecahydro-1H-cyclopenta[a]phenanthren-5-yl acetate
14	1-{1,3a,3b,5a,7-pentahydroxy-9a,11a-dimethyl-11-[(3-phenylprop-2-enoyl)oxy]-1H,2H,3H,3aH,3bH,5aH,6H,7H,8H,9H,9aH,9bH,10H,11H,11aH-cyclopenta[a]phenanthren-1-yl}ethyl benzoate	104	4-(3a-hydroxy-15a,17a-dimethyl-3a,3b,4,5,6a,10,10a,14a,15,15a,15b,16,17,17a-tetradecahydro-3H,7aH,9H-[1,3]dioxolo[4',5':3,4]pyrano[2,3-b]cyclopenta[7,8]phenanthro[3,2-e][1,4]dioxin-1-yl)furan-2(5H)-one
15	Astragaloside A	105	Astragaloside III
16	Kahiricoside V	106	Kahiricoside III
17	Astrasieversianin Xvi	107	Cyclofoetoside B
18	Brachyoside C	108	Oleifolioside B
19	Brachyoside A	109	Trojanoside H
20	Hypolide	110	Toonaciliatavarin G
21	Trojanoside C	111	Trojanoside K

22	Dyscusin C	112	Rubescin A
23	Norethynodrel	113	17-Beta-Estradiol 3-Glucosiduronic Acid
24	Beesioside A	114	Triptoquinone C
25	Hyptadienic Acid	115	Royleanonic Acid
26	beta,4-Dimethoxybenzeneethanol	116	Withanolide 2
27	Alstonic Acid A	117	Zythiostromic Acid B
28	Zygadenine	118	Zythiostromic Acid A
29	Trojanoside J	119	Colossolactone I
30	Methylnissolin-3-O-glucoside	120	Sucrose
31	L-Leucine	121	Trigonelline HCl
32	Vanillin	122	Nicotinic acid
33	2-Pyrrolidinecarboxylic acid	123	Artemisinic acid
34	β-Elemonic acid	124	Tectoridin
35	Asarylaldehyde	125	α-Boswellic acid
36	Isosakuranetin	126	Isofraxidin
37	2-Hydroxy-4-methoxybenzaldehyde	127	Isomucronulatol
38	Baicalin	128	Stachydrine
39	(2S,3R,4S,5S,6R)-2-((2-((2R,5S)-5-((2aR,3R,4S,5aS,5bS,7S,7aR,9S,11aR,12aS)-4,7-dihydroxy-2a,5a,8,8-tetramethyl-9-(((2S,3R,4S,5R)-3,4,5-trihydroxytetrahydro-2H-pyran-2-yl)oxy)tetradecahydro-1H,12H-cyclopenta[a]cyclopropa[e]phenanthren-3-yl)-5-methyltetrahydrofuran-2-yl)propan-2-yl)oxy)-6-(hydroxymethyl)tetrahydro-2H-pyran-3,4,5-triol	129	(2aR,3R,4S,5aS,5bS,7aR,9S,11aR,12aS)-4-hydroxy-2a,5a,8,8-tetramethyl-3-((2R,5R)-6-methyl-5-(((2S,3R,4S,5S,6R)-3,4,5-trihydroxy-6-(hydroxymethyl)tetrahydro-2H-pyran-2-yl)oxy)-6-(((2S,3R,4S,5R)-3,4,5-trihydroxytetrahydro-2H-pyran-2-yl)oxy)heptan-2-yl)-9-(((2R,3R,4R,5R,6S)-3,4,5-trihydroxy-6-methyltetrahydro-2H-pyran-2-yl)oxy)dodecahydro-1H,12H-cyclopenta[a]cyclopropa[e]phenanthren-7(7aH)-one
40	1-(3a,10,11-trihydroxy-7-{[4-methoxy-5-({4-methoxy-6-methyl-5-[(3,4,5-trihydroxy-6-methyloxan-2-yl)oxy]oxan-2-yl}oxy)-6-methyloxan-2-yl]oxy}-9a,11a-dimethyl-1H,2H,3H,3aH,3bH,4H,6H,7H,8H,9H,9aH,9bH,10H,11H,11aH-cyclopenta[a]phenanthren-1-yl)ethan-1-one	130	6-({[3,4-dihydroxy-6-(hydroxymethyl)-5-[(3,4,5-trihydroxy-6-methyloxan-2-yl)oxy]oxan-2-yl]oxy}methyl)-3,4,5-trihydroxyoxan-2-yl 10-hydroxy-2,2,6b,9,9,12a,14b-heptamethyl-1,2,3,4,4a,5,6,6b,7,8,8a,9,10,11,12,12a,12b,13,14,14b-icosahydropicene-4a-carboxylate
41	3-methyl-6-(1-{4,7,10-trihydroxy-3a,6,6,9a,11a-pentamethyl-1H,2H,3H,3aH,4H,5H,5aH,6H,7H,8H,9H,9aH,10H,11H,11aH-cyclopenta[a]phenanthren-1-yl}ethyl)oxan-2-one	131	(1aS,2S,4aR,4bR,6aR,7R,8R,9R,9aS,9bS,11R,11aS)-7-((2R,6S)-7-hydroxy-6-methylheptan-2-yl)-4a,6a-dimethyl-2-(((2S,3R,4S,5R)-3,4,5-trihydroxytetrahydro-2H-pyran-2-yl)oxy)tetradecahydrocyclopenta[7,8]phenanthro[1,10a-b]oxirene-8,9,9b,11(1aH)-tetraol
42	4a,6a-dimethyl-2-oxohexadecahydroindeno[5,4-f]chromen-7-yl acetate	132	7-ethenyl-2-hydroxy-1-(hydroxymethyl)-1,4b,7-trimethyl-1,2,3,4b,5,6,7,8,8a,9,10,10a-dodecahydrophenanthren-3-one
43	9,11-diacetoxy-2a,5a,8-trimethyl-3-(6-methyl-5-methyleneheptan-2-	133	5-hydroxy-4-(hydroxymethyl)-12,13,14-trimethoxy-9-oxo-3,8-

	yl)tetradecahydro-1H,12H-cyclopenta[a]cyclopropa[e]phenanthrene-8-carboxylic acid		dioxatricyclo[8.4.0.02,?]tetradeca-1(14),10,12-trien-6-yl 3,4,5-trihydroxybenzoate
44	(2R,3R,4S,5S,6R)-2-(((2S,3R,4S,5R)-4,5-dihydroxy-2-(((2aR,3R,4S,5aS,5bR,7S,7aS,9S,11aR,12aS)-4-hydroxy-3-((2R,5R)-5-(2-hydroxypropan-2-yl)-2-methyltetrahydrofuran-2-yl)-2a,5a,8,8-tetramethyl-7-(((2S,3R,4S,5S,6R)-3,4,5-trihydroxy-6-(hydroxymethyl)tetrahydro-2H-pyran-2-yl)oxy)tetradecahydro-1H,12H-cyclopenta[a]cyclopropa[e]phenanthren-9-yl)oxy)tetrahydro-2H-pyran-3-yl)oxy)-6-(hydroxymethyl)tetrahydro-2H-pyran-3,4,5-triol	134	(2R,3R,4S,5R,6R)-2-(((2aR,3R,4S,5aS,5bS,7S,7aR,9S,11aR,12aS)-4-hydroxy-3-((2R,5S)-6-hydroxy-6-methyl-5-(((2S,3R,4S,5R,6R)-3,4,5-trihydroxy-6-(hydroxymethyl)tetrahydro-2H-pyran-2-yl)oxy)heptan-2-yl)-2a,5a,8,8-tetramethyl-9-(((2S,3R,4R,5R)-3,4,5-trihydroxytetrahydro-2H-pyran-2-yl)oxy)tetradecahydro-1H,12H-cyclopenta[a]cyclopropa[e]phenanthren-7-yl)oxy)-6-(hydroxymethyl)tetrahydro-2H-pyran-3,4,5-triol
45	(2aR,4'R,6aR,7aS,9aR,9bR,10R,12S,13aR,14aS,14bS)-3,3,4',9a,10,14a-hexamethyloctadecahydro-4H,5'H,7H-spiro[cyclopropa[1',8a']naphtho[2',1':4,5]indeno[2,1-b]pyran-12,2'-furan]-4,5'-dione	135	3-(5-(2-hydroxypropan-2-yl)-2-methyltetrahydrofuran-2-yl)-2a,5a,8,8-tetramethyltetradecahydro-1H,12H-cyclopenta[a]cyclopropa[e]phenanthrene-4,7,9-triol
46	8-methoxy-11a-methyl-7-[(3,4,5,6-tetrahydroxyoxan-2-yl)oxy]-1H,2H,3H,3aH,3bH,4H,5H,9bH,10H,11H,11aH-cyclopenta[a]phenanthren-1-one	136	11-hydroxy-10-(hydroxymethyl)-2,5a,7a,7b,10,13a-hexamethyl-1,5,5a,6,7,7a,7b,8,9,9a,10,11,12,13,13a,13b-hexadecahydro-2,5-methanochryseno[2,1-c]oxepin-3(2H)-one
47	3,6-dihydroxy-9b-methyl-7-(propan-2-yl)-1H,3H,3bH,4H,5H,9bH,10H,11H-phenanthro[1,2-c]furan-1-one	137	2,3-dimethyl 4,5-bis(benzoyloxy)-7-ethenyl-8a,9-dihydroxy-1,1,4a,7-tetramethyl-8-oxo-tetradecahydrophenanthrene-2,3-dicarboxylate
48	(3aS,3a1R,5aS,6R,6aR,9S,9aR,11bR)-9-(furan-3-yl)-6-hydroxy-3a,6a,9a,11b-tetramethyl-3a,4,5a,6,6a,8,9,9a,10,11b-decahydrocyclopenta[7,8]phenanthro[10,1-bc]furan-1(3a1H)-one	138	1-{7-hydroxy-9a,11a-dimethyl-1H,2H,3H,3aH,3bH,4H,6H,7H,8H,9H,9aH,9bH,10H,11H,11aH-cyclopenta[a]phenanthren-1-yl}ethyl 3-(4-hydroxyphenyl)prop-2-enoate
49	5,6-dihydroxy-9b-methyl-7-(propan-2-yl)-1H,3H,3bH,4H,5H,9bH,10H,11H-phenanthro[1,2-c]furan-1-one	139	(2R,3R,4S,4aS,10bS)-3-hydroxy-2-(hydroxymethyl)-8,9,10-trimethoxy-6-oxo-2,3,4,4a,6,10b-hexahydropyrano[3,2-c]isochromen-4-yl 3,4,5-trihydroxybenzoate
50	1-(1,2-dihydroxyethyl)-9a,11a-dimethyl-1H,2H,3H,3aH,3bH,7H,8H,9H,9aH,9bH,10H,11H,11aH-cyclopenta[a]phenanthren-7-one	140	1-{3a,3b,7-trihydroxy-9a,11a-dimethyl-1H,2H,3H,3aH,3bH,4H,6H,7H,8H,9H,9aH,9bH,10H,11H,11aH-cyclopenta[a]phenanthren-1-yl}ethan-1-one
51	3,10-dihydroxy-4,7,11,12b,14a-pentamethyl-2,8,8a,9,10,11,12,12a,12b,13,14,14a-dodecahydropicene-2,9-dione	141	1-{7-hydroxy-9a,11a-dimethyl-1H,2H,3H,3aH,3bH,4H,6H,7H,8H,9H,9aH,9bH,10H,11H,11aH-cyclopenta[a]phenanthren-1-yl}ethyl 3-(4-hydroxyphenyl)prop-2-enoate

52	1-(furan-3-yl)-1,8-dihydroxy-3b,6,6,9a,11a-pentamethyl-1H,2H,3bH,4H,6H,7H,9aH,9bH,10H,11H,11aH-cyclopenta[a]phenanthrene-4,7-dione	142	3'-(1-hydroxy-2,3,4-trimethylpentyl)-3',9a,11a-trimethyl-2,3,3a,3b,4,6,7,8,9,9a,9b,10,11,11a-tetradecahydrospiro[cyclopenta[a]phenanthrene-1,2'-oxiran]-7-ol
53	Corticosterone	143	1,10-dihydroxy-9a,11a-dimethyl-7-oxo-1H,2H,3H,3aH,3bH,4H,5H,7H,9aH,9bH,10H,11H,11aH-cyclopenta[a]phenanthrene-1-carboxylic acid
54	11a-methyl-hexadecahydro-1H-cyclopenta[a]phenanthrene-2,7-diol	144	(24E)-3,4-Hydroxycucurbita-5,24-Diene-26-Oic Acid
55	7,10-bis(acetyloxy)-1-ethynyl-11a-methyl-1H,2H,3H,3aH,3bH,4H,5H,9bH,10H,11H,11aH-cyclopenta[a]phenanthren-1-yl acetate	145	(2S,4aS,4bS,7R)-2,4b,7-trihydroxy-8,8-dimethyl-1,2,3,4,4a,4b,5,6,7,8,8a,9-dodecahydrophenanthrene-1-carboxylic acid
56	10-(2H-1,3-benzodioxol-5-yl)-1-hydroxy-11a-methyl-1-(prop-1-yn-1-yl)-1H,2H,3H,3aH,3bH,4H,5H,7H,8H,9H,10H,11H,11aH-cyclopenta[a]phenanthren-7-one	146	4,9-dihydroxy-3,3,6b,8a,9,13a-hexamethyl-11-(2-methylprop-1-en-1-yl)-3,4,5,6,6a,6b,8,8a,8b,9,10,11,12a,13,13a,13b-hexadecahydronaphtho[2',1':4,5]indeno[2,1-b]pyran-7(1H)-one
57	3,8,9,10-tetrahydroxy-2-(hydroxymethyl)-6-oxo-1H,2H,3H,4H,4aH,6H,10bH-benzo[c]chromen-4-yl 4-hydroxybenzoate	147	1-acetyl-9a,11a-dimethyl-hexadecahydro-1H-cyclopenta[a]phenanthren-7-yl 2-(4-benzoylphenyl)acetate
58	4-hydroxy-2-methyl-6-(2a,5a,8,8-tetramethyl-9-oxotetradecahydro-1H,12H-cyclopenta[a]cyclopropa[e]phenanthren-3-yl)hept-2-enoic acid	148	1-(1,2-dihydroxyethyl)-10-hydroxy-9a,11a-dimethyl-1H,2H,3H,3aH,3bH,4H,5H,7H,8H,9H,9aH,9bH,10H,11H,11aH-cyclopenta[a]phenanthren-7-one
59	12'-(furan-3-yl)-8'-hydroxy-3-(hydroxymethyl)-6',6',8a',12a'-tetramethyloctahydro-1'H,3'H,4a'H-spiro[cyclopentane-1,4'-oxireno[2,3-d]pyrano[4',3':3,3a]isobenzofuro[5,4-f]isochromene]-3',7',10'(8'H,9a'H)-trione	149	(2S,3R,4S,5R)-2-(((2aR,3R,4S,5aS,5bS,7S,7aR,9S,11aR,12aS)-3-((2R,5S)-5,6-dihydroxy-6-methylheptan-2-yl)-4,9-dihydroxy-2a,5a,8,8-tetramethyltetradecahydro-1H,12H-cyclopenta[a]cyclopropa[e]phenanthren-7-yl)oxy)tetrahydro-2H-pyran-3,4,5-triol
60	Cyclocanthoside E	150	Cyclocanthoside D
61	Kahiricoside Iv	151	Astragaloside II
62	Indioside J	152	Astragaloside Vii
63	Oleifolioside A	153	Askendoside G
64	Trojanoside B	154	Asernestioside C
65	Trojanoside A	155	Trojanoside I
66	Astraverrucin V	156	Astraverrucin Vi
67	Mangiferonic Acid	157	Dehydrotumulosic Acid
68	Asparagoside A	158	Tupisteroide A
69	Triptoquinone A	159	Dyscusin C
70	Physalin T	160	Hoodigoside X
71	Cyclocanthoside A	161	Sutherlandioside B
72	Arbusculic Acid A	162	Carnosifloside III

73	Platanic Acid	163	Siphonol E
74	Balsaminapentaol A	164	Trojanoside K
75	Ononin	165	α-Linolenic acid
76	Ferulic acid	166	Formononetin
77	Genistin	167	5-Hydroxymethylfurfura
78	Isoguanosine	168	3,5-Dimethoxy-4-hydroxybenzaldehyde
79	Wilforlide A	169	Complanatuside
80	Tectorigenin	170	7-Hydroxycoumarin
81	4',7-Di-O-methylnaringenin	171	Dihydroartemisinin
82	Isomucronulatol 7-O-glucoside	172	Azelaic acid
83	Phloridzin	173	hederagenin
84	(2S,3R,4S,5R)-2-(((2aR,3R,4S,5aS,5bR,7S,7aS,9S,11aR,12aS)-4-hydroxy-3-((2R,5S)-5-(2-hydroxypropan-2-yl)-2-methyltetrahydrofuran-2-yl)-2a,5a,8,8-tetramethyl-7-(((2R,3R,4S,5S,6R)-3,4,5-trihydroxy-6-(hydroxymethyl)tetrahydro-2H-pyran-2-yl)oxy)tetradecahydro-1H,12H-cyclopenta[a]cyclopropa[e]phenanthren-9-yl)oxy)tetrahydro-2H-pyran-3,4,5-triyl triacetate	174	(2aR,3R,4S,5aS,5bS,7S,7aR,9S,11aR,12aS)-3-((2R,5R)-5-hydroxy-6-methyl-6-(((2S,3R,4S,5R)-3,4,5-trihydroxytetrahydro-2H-pyran-2-yl)oxy)heptan-2-yl)-2a,5a,8,8-tetramethyl-7,9-bis(((2R,3R,4R,5R,6S)-3,4,5-trihydroxy-6-methyltetrahydro-2H-pyran-2-yl)oxy)tetradecahydro-1H,12H-cyclopenta[a]cyclopropa[e]phenanthren-4-yl acetate
85	7-[(4-hydroxy-5-{[5-({4-hydroxy-5-[(5-hydroxy-4-methoxy-6-methyloxan-2-yl)oxy]-6-methyloxan-2-yl}oxy)-4-methoxy-6-methyloxan-2-yl]oxy}-6-methyloxan-2-yl)oxy]-1-(1-hydroxyethyl)-9a,11a-dimethyl-1H,2H,3H,3aH,3bH,4H,6H,7H,8H,9H,9aH,9bH,10H,11H,11aH-cyclopenta[a]phenanthrene-1,3a,3b,11-tetrol	175	Calycosin
86	4-(3a,11-dihydroxy-7-{[4-hydroxy-5-({4-hydroxy-5-[(4-hydroxy-6-methyl-5-{[2,3,4-trihydroxy-5-(hydroxymethyl)cyclohexyl]oxy}oxan-2-yl)oxy]-6-methyloxan-2-yl}oxy)-6-methyloxan-2-yl]oxy}-9a,11a-dimethyl-hexadecahydro-1H-cyclopenta[a]phenanthren-1-yl)-2,5-dihydrofuran-2-one	176	Calycosin-7-O-β-D-glucoside
87	11a-methyl-7-oxo-1H,2H,3H,3aH,3bH,4H,5H,7H,8H,9H,9aH,9bH,10H,11H,11aH-cyclopenta[a]phenanthren-1-yl acetate	177	10-hydroxy-1-(2-hydroxyacetyl)-11a-methyl-1H,2H,3H,3aH,3bH,4H,5H,7H,8H,9H,9aH,9bH,10H,11H,11aH-cyclopenta[a]phenanthren-7-one
88	9a,11a-dimethyl-1-[1-(methylamino)ethyl]-1H,2H,3H,3aH,3bH,4H,6H,7H,8H,9H,9aH,9bH,10H,11H,11aH-cyclopenta[a]phenanthrene-2,7-diol	178	(1R,3aS,3bR,6aR,7aS,9R,11aR,12aR,13aS,13bS,15aR)-3a,11a-dihydroxy-9,15a-dimethyl-11-oxo-1-(5-oxo-2,5-dihydrofuran-3-yl)-1,2,3,3a,3b,4,6,6a,7a,10,11,11a,12a,13,13b,1

			4,15,15a-octadecahydro-9H,13aH-cyclopenta[7,8]phenanthro[2,3-b]pyrano[3,2-e][1,4]dioxine-13a-carbaldehyd
89	2-((1R,3aS,5aR,5bR,7aR,10R,10aR,10bR,12R,12aS,12bS)-7a-((((2S,3R,4S,5S,6R)-6-((((2R,3R,4R,5S,6R)-3,4-dihydroxy-6-(hydroxymethyl)-5-(((2S,3R,4R,5R,6S)-3,4,5-trihydroxy-6-methyltetrahydro-2H-pyran-2-yl)oxy)tetrahydro-2H-pyran-2-yl)oxy)methyl)-3,4,5-trihydroxytetrahydro-2H-pyran-2-yl)oxy)carbonyl)-12-hydroxy-3,3,5a,5b,12b-pentamethyl-10-(prop-1-en-2-yl)octadecahydro-1H-cyclopenta[7,8]phenanthro[1,2-c]furan-1-yl)acetic acid	179	3-((4,5-diacetoxy-6-((4-hydroxy-3-(5-(2-hydroxypropan-2-yl)-2-methyltetrahydrofuran-2-yl)-2a,5a,8,8-tetramethyl-7-((3,4,5-trihydroxy-6-(hydroxymethyl)tetrahydro-2H-pyran-2-yl)oxy)tetradecahydro-1H,12H-cyclopenta[a]cyclopropa[e]phenanthren-9-yl)oxy)tetrahydro-2H-pyran-3-yl)oxy)-3-oxopropanoic acid
90	2-{[2-(5-{9,14-dihydroxy-7,7,12,16-tetramethyl-6-[(3,4,5-trihydroxyoxan-2-yl)oxy]pentacyclooctadecan-15-yl}-5-methyloxolan-2-yl)propan-2-yl]oxy-6-(hydroxymethyl)		

2. Supplemental Figures

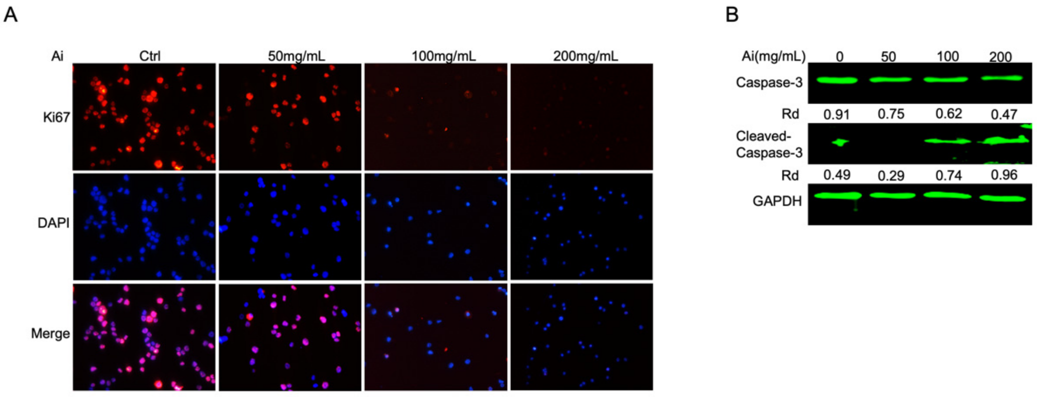

Figure S1. Inhibition of cell proliferation and apoptosis by Ai in HEL cells. (A) Inhibition of proliferation of HEL cells by Ai is monitored through staining with anti-Ki67. DAPI and merge with Ki67 is shown in nucleus. (B) Treatment with Ai induces apoptosis associated with caspase-3 cleavages. GAPDH is used as loading control.

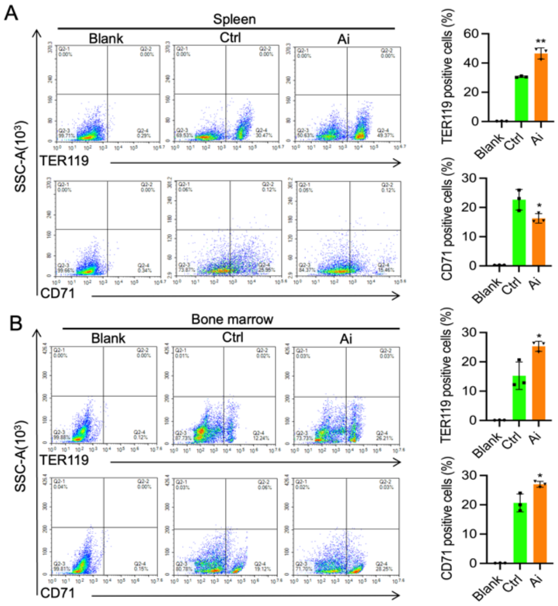

Figure S2. Higher erythroid cells in leukemic mice treated by Ai. (A-B) Flow cytometry analysis of spleen (A) and bone marrow (B) of control and Ai treated mice for expression of erythroid markers TER119 and CD71. Data are expressed as mean ± SD, n = 3 independent experiments. ** $p < 0.01$ and * $p < 0.05$.

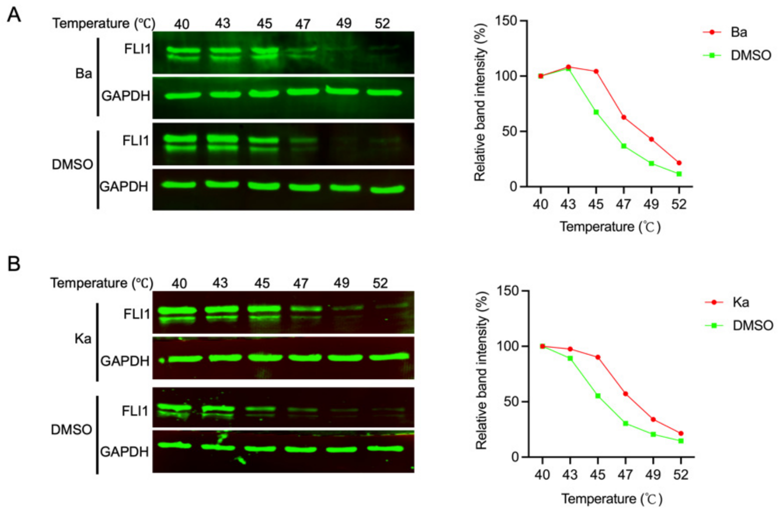

Figure S3. Protein stability of the FLI1 in HEL cells after treatment with Ba and Ka. (A-B) The binding ability of Ba (A) and Ka (B) to FLI1 protein was tested in cellular thermal shift assay (CETSA) by western blotting. The value of the band quantified by densitometry shown in the right panel.

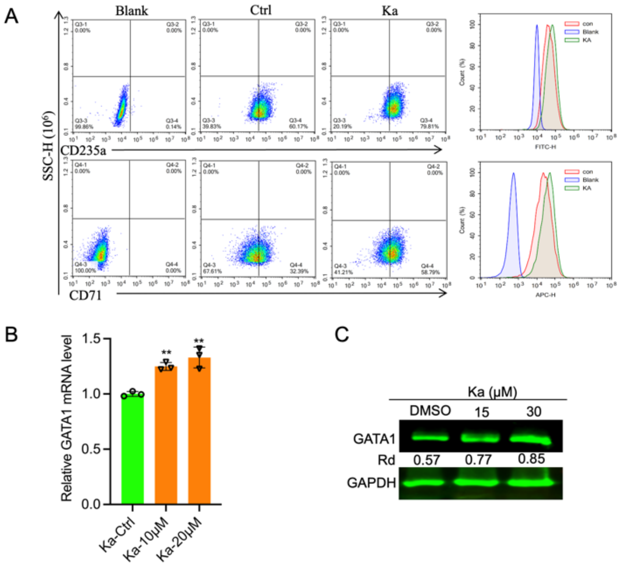

Figure S4. Ka induces differentiation of HEL cells in culture. (A) Ka treatment (30uM) Increases the percentage of CD235a/CD71 erythroid markers in HEL cells, determined by flow cytometry. (B-C) Ka treatment of HEL cells increases GATA1 mRNA (B) and protein (C) level, determine by RT-qPCR and western blotting, respectively. Data are expressed as mean ± SD, n = 3 independent experiments. ** *p* < 0.01.

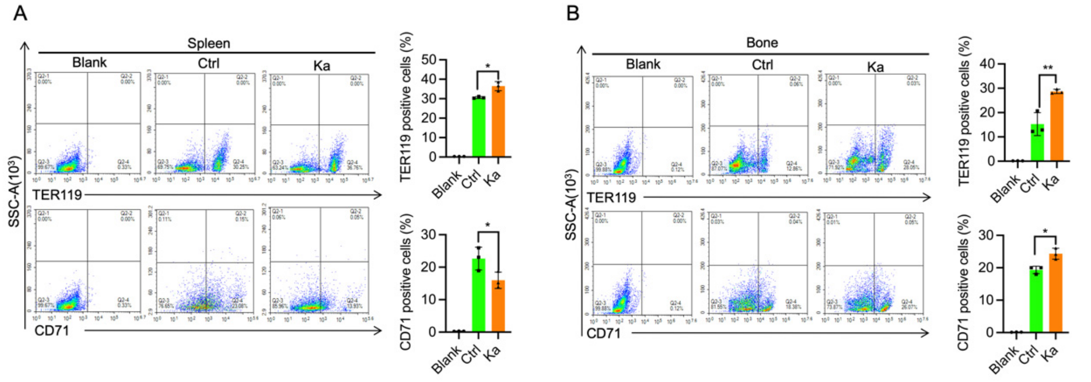

Figure S5. Increase erythroid cells in leukemic mice treated by Ka versus control. (A-B) Flow cytometry analysis of spleen (A) and bone marrow (B) of control and Ai treated mice for expression of erythroid markers TER119 and CD71. Data are expressed as mean ± SD, n = 3 independent experiments. ** $p < 0.01$ and * $p < 0.05$.

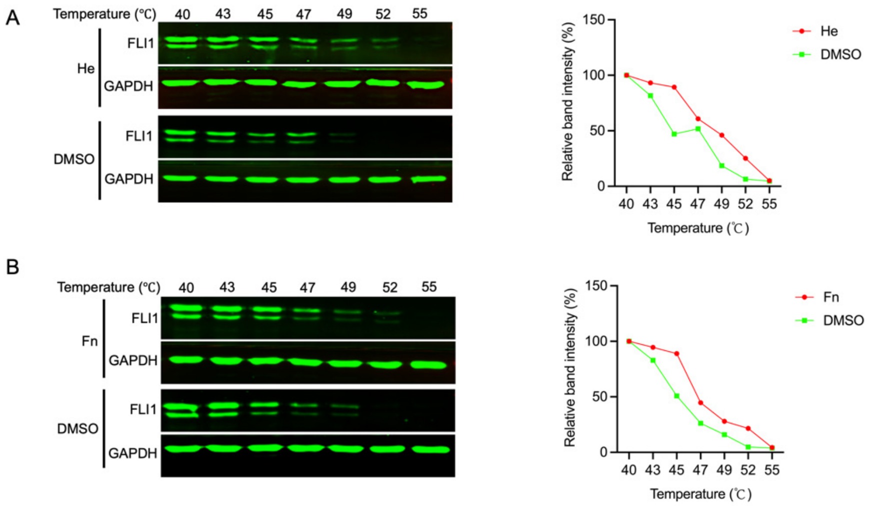

Figure S6. Protein stability of the FLI1 in HEL cells after treatment with anti-FLI1 compounds He and Fn. (A-B) The binding ability of He (A) and Fn (B) to FLI1 protein was tested in cellular thermal shift assay (CETSA) by western blotting. The value of the band quantified by densitometry shown in the right panel.

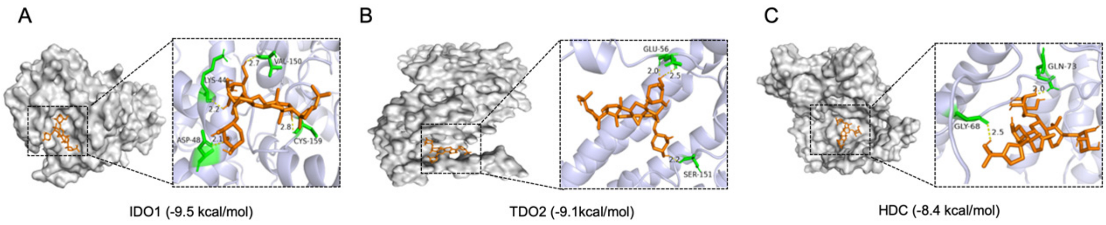

Figure S7. Three- and two-dimensional interaction of AS-A compound to IDO1 (A), TDO2 (B) and HDC (C). Binding energy (kcal/mol) for each gene was shown under.

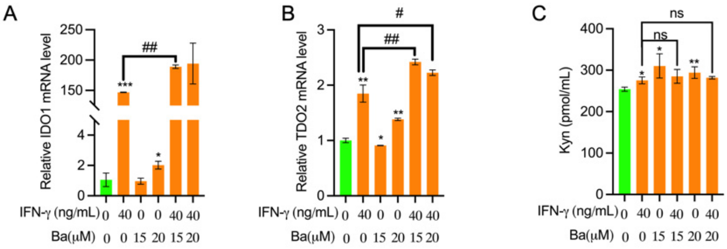

Figure S8. Effect of anti-FLI1 compound Betulinic acid on tryptophan metabolism in leukemic cells. (A,B) Expression of IDO1 (A) and TDO2 (B) in HEL cells treated with Ba (15 and 20μM) and Ba+IFN-γ, as determined by RT-qPCR. (C) Kyn expression in HEL cells treated with Ba and Ba+IFN-γ, as determined by Elisa assay. Data are expressed as mean ± SD, n = 3 independent experiments. ***p<0.001, ** p < 0.01 and * p < 0.05 versus Ctrl groups; ##p < 0.01 and #p < 0.05 versus IFN-γ groups.

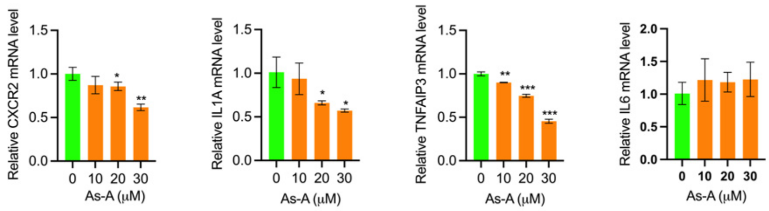

Figure S9. Expression of the CXCR2, IL1A, TNFAIP3 and IL6 genes in HEL cells treated for 24h with indicated doses of AS-A, determined by RT-qPCR. Data are expressed as mean ± SD, n = 3 independent experiments. ***p<0.001, ** p < 0.01 and * p < 0.05.
